# Practical Departments

**Published:** 1897-05-29

**Authors:** 


### PRACTICAL DEPARTMENTS.

### FISH FOR INSTITUTIONS.

(Messrs. J. and G. Woods, Fish Merchants, Grimsby.)

The provision of fish is frequently a difficulty in institutions. On one day in the week fish is usually served for dinner, and it must be admitted that this substitute for meat is not popular. Sometimes the objection is justified by unappetising cooking or the inferior quality or condition of the fish. Both objections are capable of removal in well-conducted institutions. The cooking we need not enter into here. The supply of good fish, we would point out, should be no difficulty. It is only necessary to find merchants of good standing in the fish trade, such as Messrs. Woods, and place the matter in his hands. He is willing to supply excellent fish at trade prices, and to send it so packed as to ensure its arrival fresh and in good condition. We have personally tested the quality and condition of the fish, and noted the excellent way in which it is packed in ice, which makes it possible to leave a prudent margin between the projected time of arrival and cooking to allow for accidental delay in delivery. This possible delay in the receipt of the commodity on which the whole of a large institution may be dependent for dinner frequently is the cause why contracts of a far from advantageous character are entered upon. In dealing with Messrs. Woods security may be felt, as the firm take great pains to secure prompt delivery, and as we said before the excellent packing preserves the fish fresh and wholesome for a considerable time. We cannot do better than recommend the managers of institutions to secure their fish supply through their medium.

